# Sheet-on-belt branched TiO_2_(B)/rGO powders with enhanced photocatalytic activity

**DOI:** 10.3762/bjnano.9.146

**Published:** 2018-05-24

**Authors:** Huan Xing, Wei Wen, Jin-Ming Wu

**Affiliations:** 1 State Key Laboratory of Silicon Materials, and School of Materials Science and Engineering, Zhejiang University, Hangzhou 310027, P. R. China; 2 College of Mechanical and Electrical Engineering, Hainan University, Haikou 570228, P. R. China

**Keywords:** branched nanostructure, photocatalysis, reduced graphene oxide, TiO_2_(B)

## Abstract

TiO_2_(B) is usually adopted to construct phase junctions with anatase TiO_2_ for applications in photocatalysis to facilitate charge separation; its intrinsic photocatalytic activity, especially when in the form of one- or three-dimensional nanostructures, has been rarely reported. In this study, a sheet-on-belt branched TiO_2_(B) powder was synthesized with the simultaneous incorporation of reduced graphene oxide (rGO). The monophase, hierarchically nanostructured TiO_2_(B) exhibited a reaction rate constant 1.7 times that of TiO_2_(B)/rGO and 2.9 times that of pristine TiO_2_(B) nanobelts when utilized to assist the photodegradation of phenol in water under UV light illumination. The enhanced photocatalytic activity can be attributed to the significantly increased surface area and enhanced charge separation.

## Introduction

Nowadays, TiO_2_ has found wide-spread application in energy and environmental fields because of its environmental friendliness, excellent stability, and low cost [1–8]. Among various TiO_2_ phases, TiO_2_(B) (where “B” stands for bronze, by analogy with the tungsten bronze compounds) is less common but still draws much attention. Many types of TiO_2_(B) nanostructures have been synthesized, such as nanowires [9–10], nanotubes [11], nanobelts [12–14], nanofibers [15] and nanosheets [16]. TiO_2_(B) is mostly used in lithium-ion batteries due to its relatively open crystal structure, superior safety and rate capability [11,17–20]. For photocatalytic applications, TiO_2_(B) is usually combined with anatase TiO_2_ to construct a multiphase heterostructure to enhance charge separation and in turn the photocatalytic activity [21–25]. For example, Yang et al. synthesized anatase nanocrystals on TiO_2_(B) single-crystal fibrils by a two-step process [23]. Li et al. prepared a biphase TiO_2_ core/shell nanofiber with anatase core and TiO_2_(B) shell [24]. Kandiel et al. used a hydrothermal technique to synthesize TiO_2_(B) nanofibers simultaneously decorated with anatase nanoparticles [25].

The incorporation of graphene is effective to improve the photocatalytic activity of TiO_2_ because of the increased adsorption capacity and enhanced charge separation [26]. Meanwhile, growing branches on one-dimensional (1D) TiO_2_ makes full use of the free space on the 1D nanostructures and increases the light harvesting efficiency, which also contributes to increased photocatalytic activity [22,27]. Herein, we report a novel approach to synthesize branched TiO_2_(B) nanobelts incorporated at the same time with reduced graphene oxide (rGO). The unique sheet-on-belt nanostructure demonstrates a high specific surface area and a favored charge separation, and hence, improved photocatalytic activity.

## Results and Discussion

**Figure 1:**
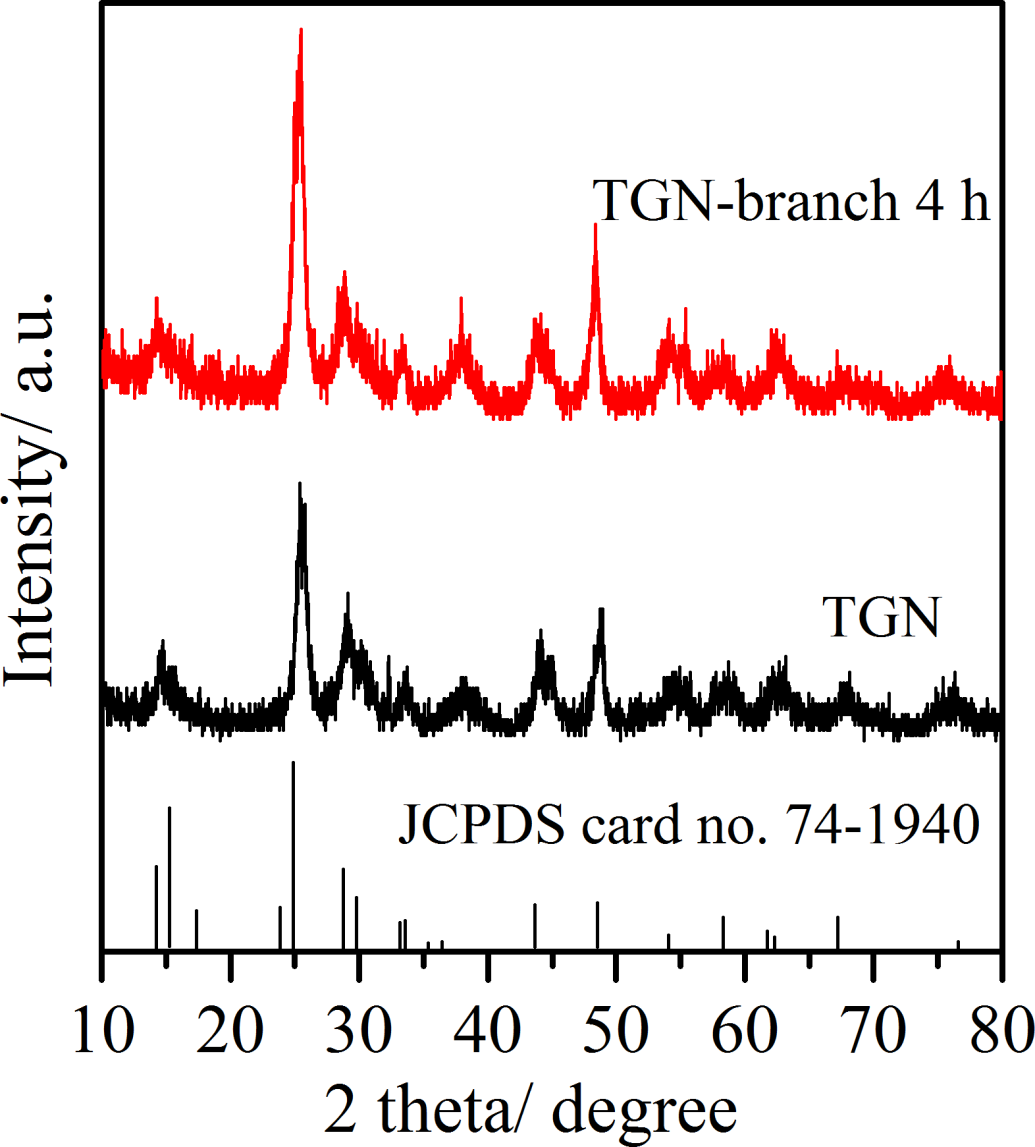
X-ray diffraction (XRD) patterns of TiO_2_(B)/rGO nanobelts (TGN) and branched TiO_2_(B)/rGO nanobelts (TGN-branch 4 h).

Figure 1 shows the X-ray diffraction (XRD) patterns of TiO_2_(B)/rGO nanobelt (designated as TGN for simplicity) and branched TiO_2_(B)/rGO nanobelt (TGN-branch 4 h, refer to the Experimental section for details of the sample ID). All Bragg diffraction peaks can be indexed to the monoclinic TiO_2_(B) phase (JCPDS card no. 74-1940) [28–30]. This suggests that both the alkali-hydrothermal synthesized TGN and the branches precipitated from the precursor solution are TiO_2_(B). No XRD peaks corresponding to graphene can be detected, which can be ascribed to its small quantity in the composite powders.

**Figure 2:**
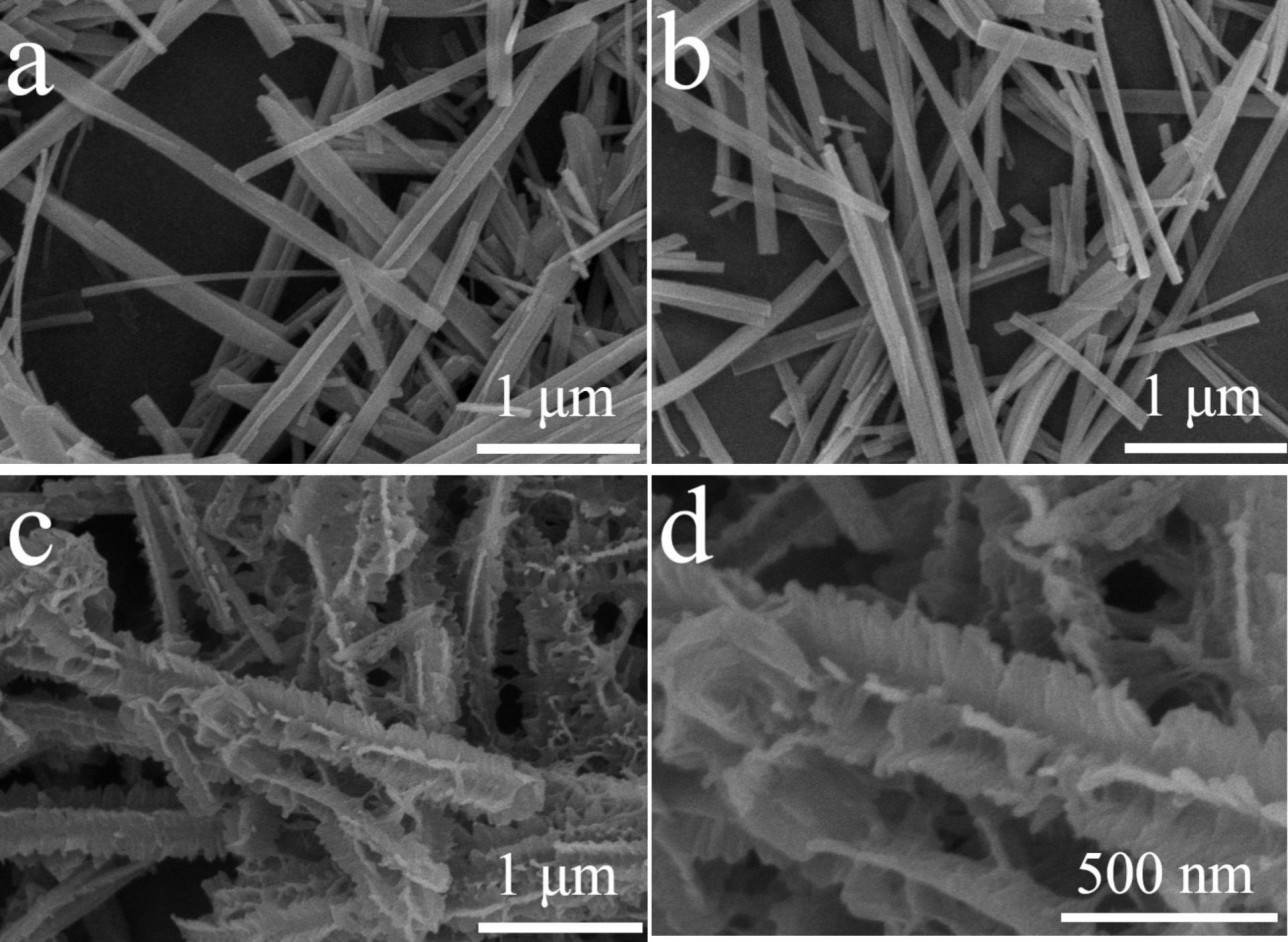
FESEM images of (a) pristine TiO_2_ nanobelts, (b) sample TGN, and (c, d) sample TGN-branch 4 h.

Figure 2 shows the field emission scanning electron microscopy (FESEM) images of the pristine TiO_2_ nanobelt structures, TGN and TGN-branch 4 h. Comparing Figure 2a and Figure 2b, no obvious difference can be discerned, suggesting that the incorporation of GO does not change the growth of TiO_2_ nanobelts during the alkali-hydrothermal procedure. The belt-like structure shows a typical size of 100–300 nm in width and several micrometers in length. Figure 2c and Figure 2d indicate that, after being immersed in the precursor solution for 4 h, the TGN was uniformly covered with sheet-like branches, exhibiting a “sheet-on-belt” morphology. Figure S1 in Supporting Information File 1 shows the morphology evolution of the branched structure by increasing the nanosheet deposition time. After a deposition time of 2 h, TiO_2_ dots were found to be distributed homogeneously on the TGN surface. The nanosheet branches grew continuously upon further precipitation for up to 24 h.

**Figure 3:**
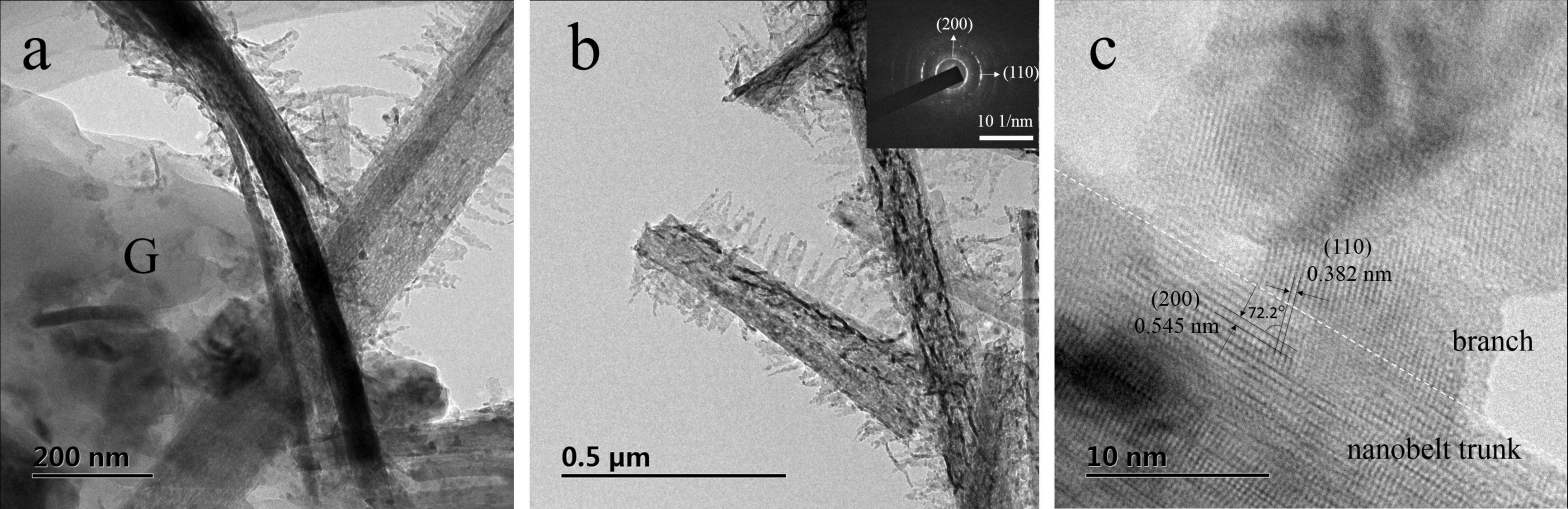
(a, b) TEM and (c) HRTEM image of sample TGN-branch 4 h. The inset in (b) shows the corresponding SAED pattern.

The transmission electron microscopy (TEM) image illustrates the precipitation of branched nanobelts on rGO surfaces (labelled as G in Figure 3a). Figure 3b shows more clearly the radially growing nanosheets along the nanobelt trunk. The average length of the nanosheet branches is ≈100 nm. The selected area electron diffraction (SAED) pattern collected from several “sheet-on-belt” nanostructures indicates diffraction rings corresponding to polycrystalline TiO_2_(B). The high-resolution TEM (HRTEM) image demonstrated in Figure 3c shows parallel fringes with a neighboring distance of ≈0.545 nm, corresponding to the (200) plane of TiO_2_(B) and distance of ≈0.382 nm, which is attributed to the (110) plane of TiO_2_(B). The cross-angle of 72.2° is in good agreement with the theoretical value between (200) and (110) planes of monoclinic TiO_2_(B).

**Figure 4:**
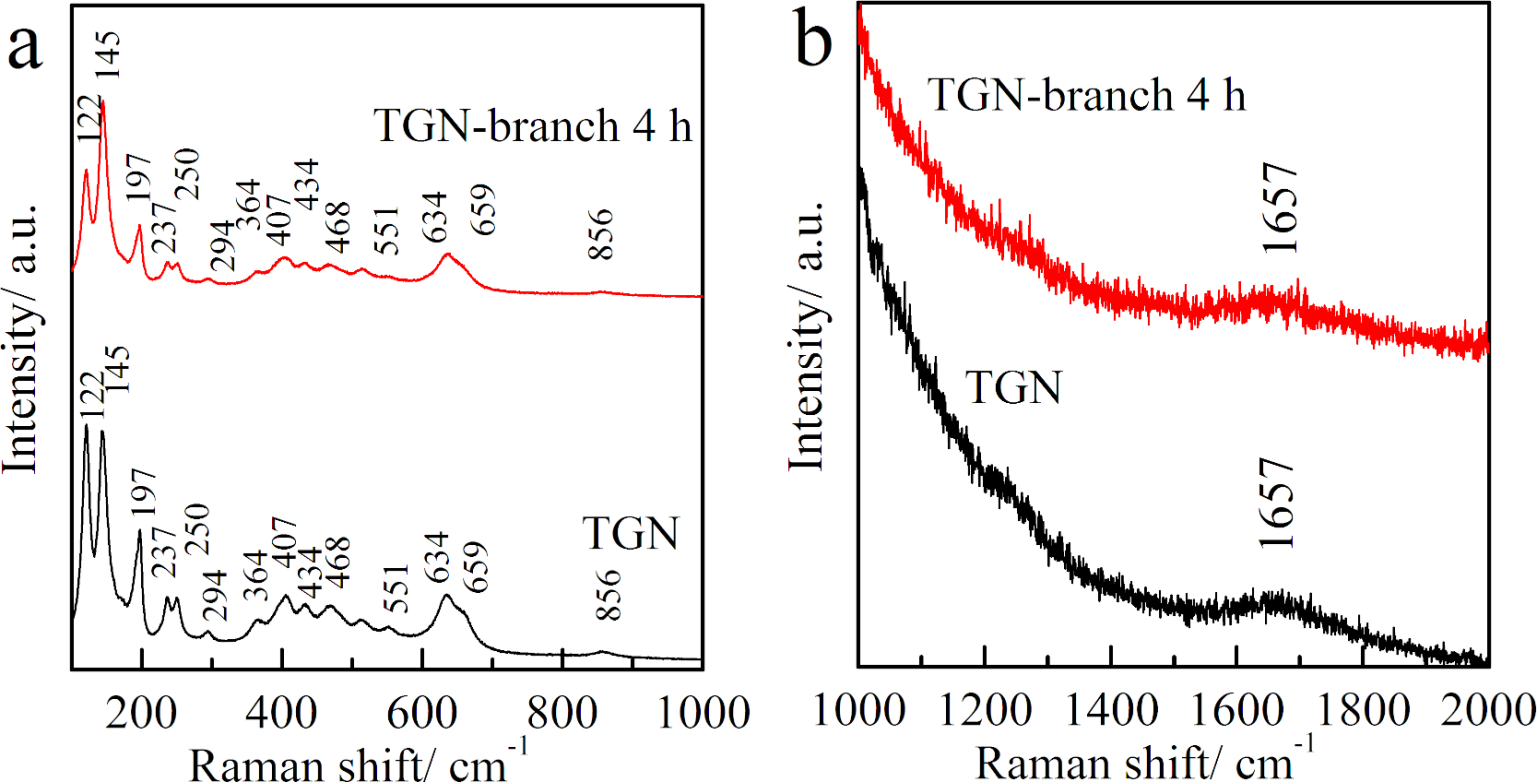
Raman spectra of samples TGN and TGN-branch 4 h recorded over the range of (a) 100–1000 cm^−1^ and (b) 1000–2000 cm^−1^.

The phase composition of samples TGN and TGN-branch 4 h was further investigated by Raman spectroscopy (Figure 4). The Raman peaks observed over the 100–1000 cm^−1^ range can be attributed to the vibrational modes of the TiO_2_(B) phase [28], which is in agreement with the XRD and HRTEM results. A weak Raman peak located at 1657 cm^−1^ can be discerned in the TGN sample, which corresponds to the G band (graphitized carbon), confirming the existence of graphene in the powders [31]. The peak intensity decreased in sample TGN-branch 4 h, which is due to the decreased ratio of rGO in TGN after growing branches.

It is reported that the nanosheet branches precipitated from the present precursor solution are few-layer hydrogen titanates [32]. When precipitated homogeneously from the solution, or precipitated heterogeneously on metallic Ti foils, such titanates decomposed to anatase TiO_2_ upon calcination in air at 400 °C for 1 h [32–33]. In the current investigation, hydrogen titanate nanosheets are supposed to nucleate heterogeneously on the TiO_2_(B) trunks via surface deficiencies or exposed ledges when immersing the pristine TiO_2_(B) in the precursor solution at 60 °C, which then grow continuously during the prolonged duration. After the final calcination at 400 °C for 1 h in air, the titanate branches decomposed to TiO_2_(B) instead of anatase TiO_2_. It thus suggests that the decomposition and crystallization of hydrogen titanates depends on the substrates. The TiO_2_(B) trunk may guide the TiO_2_ crystallization.

**Figure 5:**
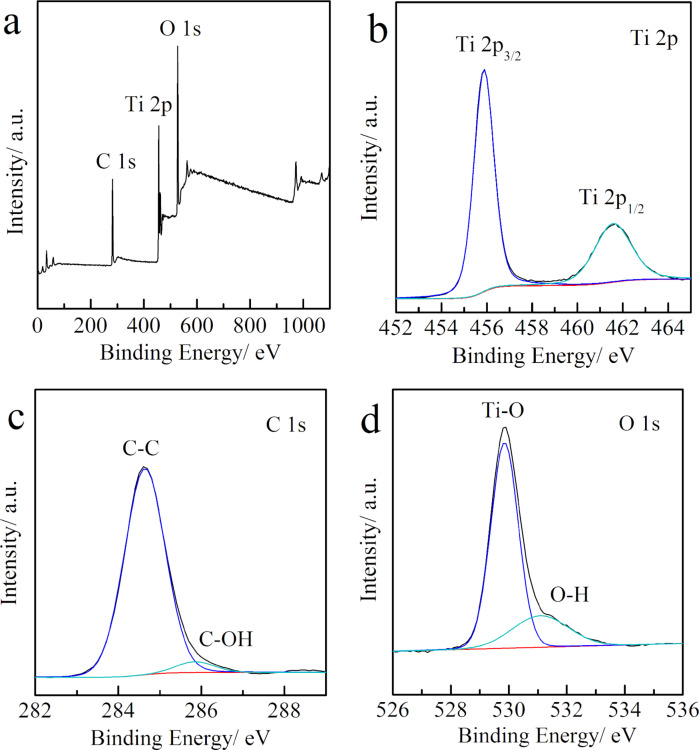
(a) XPS survey spectrum and core level XPS spectra of (b) Ti 2p, (c) C 1s, and (d) O 1s for sample TGN-branch 4 h.

The chemical bonding states of the TGN-branch 4 h were determined by X-ray photoelectron spectra (XPS). Elements of Ti, C and O have been detected in the XPS survey spectra (Figure 5a). The binding energy of Ti 2p at 455.8 eV and 461.6 eV correspond to Ti 2p_3/2_ and Ti 2p_1/2_, respectively (Figure 5b). The separation between the two peaks is 5.8 eV, which is typical of Ti^4+^ in the TiO_2_ lattice [34]. In Figure 5c, the two peaks located at 284.6 eV and 285.9 eV in the C 1s spectrum can be found, which correspond to carbon bonds of C–C and C–OH [35–36]. Two typical peaks at 529.6 eV and 531.2 eV have been observed for the O 1s spectrum in Figure 5d, which can be attributed to Ti–O and Ti–OH bonds, respectively [37].

**Figure 6:**
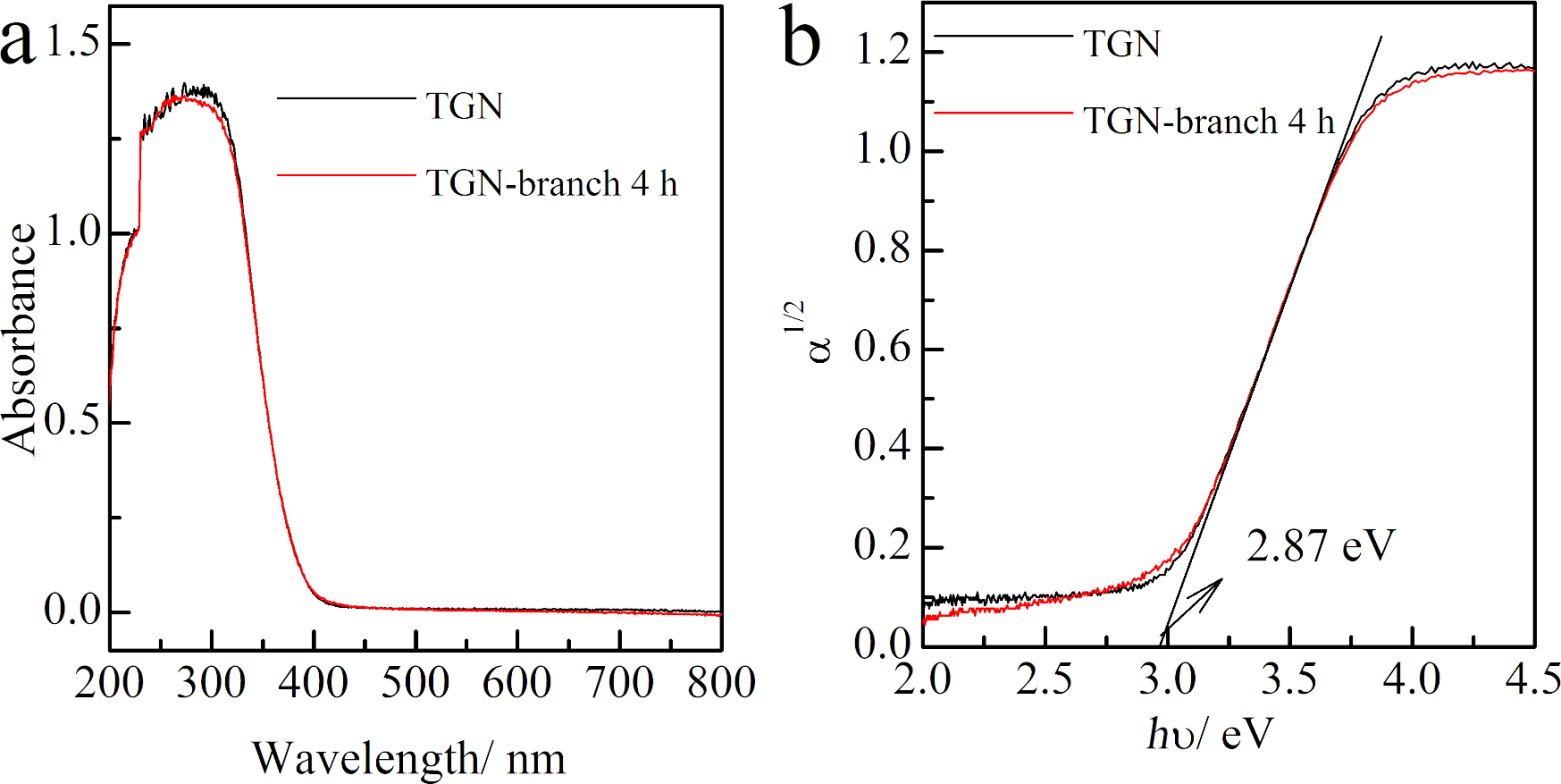
(a) UV–vis diffuse reflectance spectra of TGN and TGN-branch 4 h. (b) Re-plotting of (a) in the α^1/2^ ~ *h*ν coordinate to evaluate the corresponding band gap, assuming an indirect transition between bands for TiO_2_.

The UV–visible diffuse reflection spectra of TGN and TGN-branch 4 h are shown in Figure 6. The TGN-branch 4 h sample exhibits almost the same absorbance (Figure 6a) and the same band gap of 2.87 eV (Figure 6b) compared to TGN. This can be attributed to the same phase composition of TiO_2_(B) in both samples. The evaluated band gap of 2.87 eV for TiO_2_(B) agrees with that (2.9 eV) reported by Kandiel et al. [25]. This band gap value is smaller than that of 3.2 eV for bulk anatase TiO_2_ [27]. Chakraborty et al. also reported that the absorption band edge of the pure TiO_2_(B) is located at a relatively longer wavelength compared to that of the pure anatase TiO_2_ [21].

The low temperature nitrogen adsorption isotherm was measured to evaluate the BET specific surface area (Figure S2, Supporting Information File 1). The specific surface area of the pristine TiO_2_ nanobelt, TGN and TGN-branch 4 h powders is 30.0, 32.5 and 44.2 m^2^·g^−1^, respectively. The incorporation of rGO slightly increased the specific surface area of the pristine TiO_2_ nanobelt. The further increased surface area of the branched nanostructure can be explained by the thinner thickness (≈2 nm, Figure 2d) and thus higher specific surface area of the nanosheet branches compared with the nanobelt trunks (≈5 nm in thickness, Figure 2a and Figure 2b).

**Figure 7:**
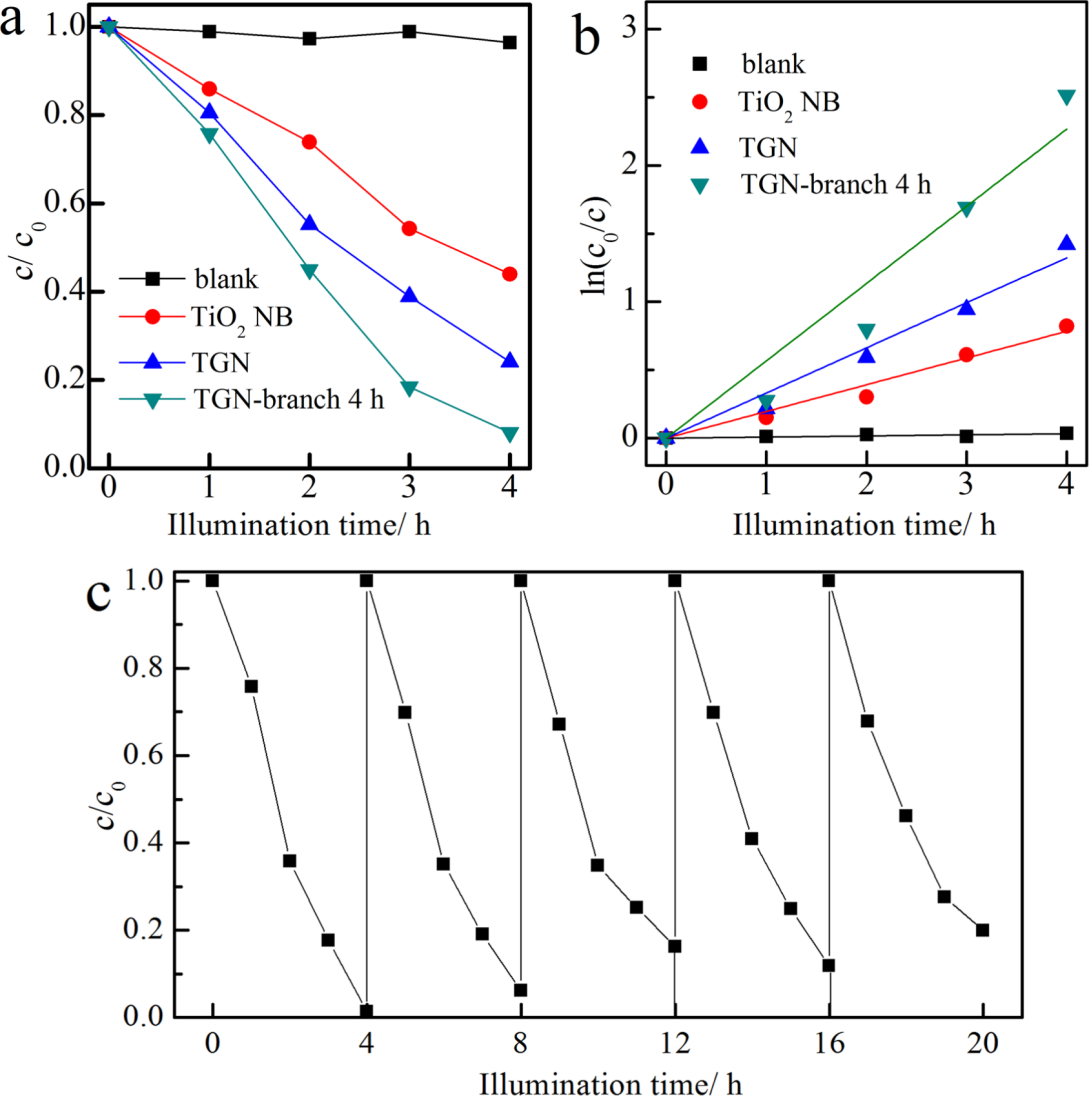
Photodegradation of phenol in the presence of TiO_2_ nanobelts (TiO_2_ NB), TGN and TGN-branch 4 h under UV light illumination: (a) the degradation curves; (b) the fitting results assuming a pseudo-first order reaction; and (c) the cycling performance of TGN-branch 4 h samples.

Photocatalytic activities of the pristine TiO_2_ nanobelt, TGN and TGN-branches were evaluated via photodegradation of phenol in water under UV light illumination. After stirring in the dark for 1 h, an adsorption–desorption equilibrium was established. About 5% phenol in water was adsorbed by all three photocatalysts. The TGN-branch 4 h sample demonstrated enhanced photocatalytic performance as compared to TiO_2_ nanobelts and the TGN sample. Figure S3 in Supporting Information File 1 shows the photodegradation curves in the presence of TGN and TGN-branches with different deposition times. All TGN-branch powders exhibited higher efficiency than TGN. The highest efficiency was achieved for the TGN-branch 4 h and TGN-branch 6 h samples, which induced over 90% phenol degradation within 4 h; whilst only 76% of phenol was degraded by TGN. For a clear demonstration, Figure 7a shows the photodegradation curves only in the presence of pristine TiO_2_ nanobelts (TiO_2_ NB), TGN and TGN-branch 4 h. Figure 7b shows an almost linear relationship between ln(*c*_0_/*c*) and the illumination time, which indicates that the photodegradation follows roughly a pseudo-first order kinetic. The reaction rate constant determined for the TGN-branch 4 h and TGN-branch 6 h samples was the same at ≈0.57 h^−1^; whilst that of the pristine TiO_2_ nanobelt and TGN samples was only 0.20 h^−1^ and 0.33 h^−1^, respectively. It concludes that the incorporation of rGO in TiO_2_(B) nanobelts increased the photocatalytic activity, which is in agreement with the numerous reports on TiO_2_/rGO hybrids, because the superior electrical conductivity of graphene improves the separation of photogenerated carriers [26,38]. Interestingly, a simple immersion of the TiO_2_(B)/rGO sample in the current precursor solution to form the “sheet-on-belt” branched nanostructures achieved significantly enhanced photocatalytic activity, which is 1.7 times that of TiO_2_(B)/rGO, and 2.9 times that of pristine TiO_2_(B) nanobelts. Figure 7c confirms the stability of TGN-branch 4 h. The photocatalytic activity remained almost unchanged by repetitively degrading phenol in water under UV light illumination for up to 5 cycles. The slightly decreased efficiency can be attributed to the catalyst loss during the centrifugation process after each cycle.

The reaction rate constants were normalized with the corresponding specific surface area of the photocatalysts to see whether or not the increasing specific surface area is the sole contribution. For the pristine TiO_2_ nanobelt, TGN and TGN-4 h samples, the normalized values are 0.0067, 0.010, and 0.013 h^−1^·g·m^−2^, respectively. This fact indicates that there are synergetic effects in the present “sheet-on-belt” branched TiO_2_(B)/rGO powders.

**Figure 8:**
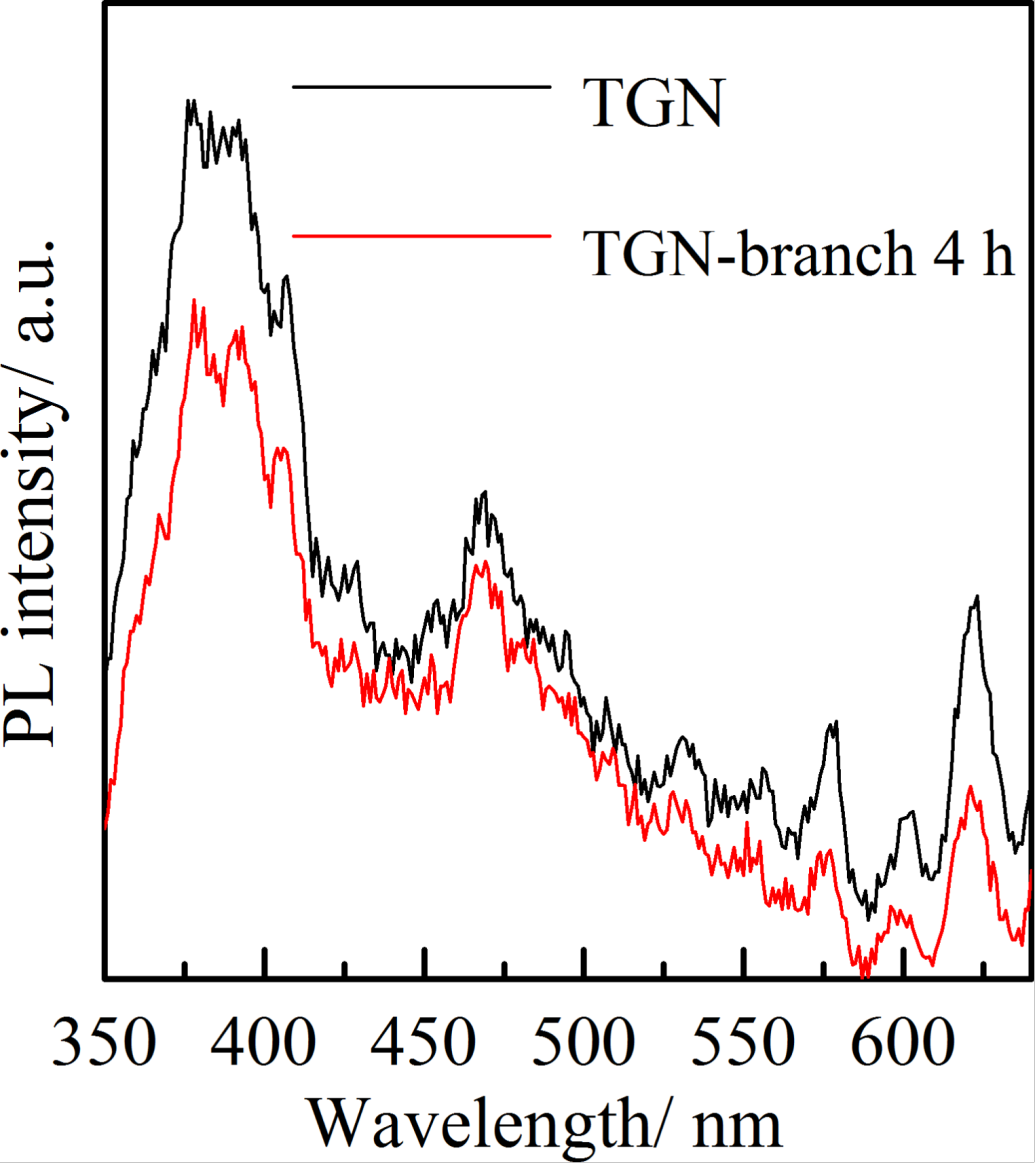
Photoluminescence spectra of TGN and TGN-branch 4 h.

Figure 8 shows photoluminescence (PL) spectra of TGN and TGN-branch 4 h samples. The PL intensity of TGN is stronger than that of TGN-branch 4 h, indicating a faster recombination of carriers in TGN. This result demonstrates that growing branches on TGN contributes to the separation of photogenerated charge carriers, and in turn, an improved photocatalytic activity [39]. In the current investigation, both trunks and branches are TiO_2_(B); however, one can still anticipate phase junctions among the interfaces. This is because the trunks and branches were synthesized by different techniques and hence are of different sizes, and especially different grain sizes that would readily affect the band gaps. Song et al. reported that the composite of anatase TiO_2_ synthesized via two different routes resulted in enhanced charge separation and hence increased photo-electrochemical response and photocatalytic activity [40].

## Conclusion

TiO_2_(B) nanosheet branches were uniformly in situ grown on TiO_2_/rGO nanobelt surfaces to form a unique sheet-on-belt nanostructure. Both trunks and branches were TiO_2_(B); however, the photoluminescence measurements suggest an enhanced charge separation of the branched nanobelts when compared with the pristine alkali-hydrothermal synthesized nanobelt. The incorporation of graphene and the branching tactic resulted in a significantly increased specific surface area. When utilized to assist photodegradation of phenol in water under UV light illumination, the reaction rate constant of the present “sheet-on-belt” branched TiO_2_(B)/rGO photocatalyst is 1.7 times that of TiO_2_(B)/rGO and 2.9 times that of pristine TiO_2_(B) nanobelts.

## Experimental

### Material synthesis

**1. Precursor solution:** The precursor solution for precipitation of nanosheet branches was prepared following Wen et al. [32]. In brief, 1.25 g of TiOSO_4_, 1.75 g of C_2_H_5_NO_2_ and 0.6 mL of HNO_3_ (65%) were dissolved in 10 mL deionized water, which was placed in a 400 °C muffle furnace for 15 min to obtain a black powder. 0.5 g of the as-synthesized black powder was immersed in 50 mL of 30 wt % H_2_O_2_ aqueous solution and maintained for 72 h at room temperature. After the reaction, the precipitate was centrifugally removed and the remaining solution served as the precursor solution.

**2. Synthesis of TiO_2_(B)/rGO nanobelts (TGN):** Graphene oxide (GO) was synthesized starting from graphite flakes by a modified Hummers' method according to our previous report [31]. To synthesize the TiO_2_(B)/rGO nanobelt (designated as TGN), 3 mL of GO suspension with a concentration of 2 mg/mL was added to 30 mL of 10 M NaOH aqueous solution, together with 0.2 g commercial Degussa P25 TiO_2_ nanoparticles. The reactant was then transferred to a Teflon-lined stainless steel autoclave and maintained at 200 °C for 24 h, with a heating rate of 2 °C/min. After the reaction, the precipitates were centrifugally washed with distilled water three times and then immersed in a 0.1 M HCl solution for 1 h repetitively for three times to complete the proton exchange. The precipitates were then dried at 60 °C and calcined at 450 °C for 1 h in air. For comparison, pristine TiO_2_ nanobelts were synthesized via the same procedure but in absence of the GO suspension.

**3. Synthesis of branched TiO_2_(B)/rGO nanobelts (TGN-branch):** 20 mg of the as-prepared TGN was dispersed in 15 mL precursor solution and maintained at 60 °C for 2–8 h. The precipitates were centrifugally washed with distilled water and ethanol each for three times respectively and dried at 60 °C, followed by a final calcination at 400 °C for 1 h in air. The resultants were termed as TGN-branch *x* h, where *x* refers to the precipitation time in the precursor solution.

### Characterization

The morphology was examined by field emission scanning electron microscopy (FESEM, Hitachi S-4800) and transmission electron microscopy (TEM, JEM-2100). The X-ray diffraction (XRD) patterns were collected using a Rigaku D/max-3B diffractometer with Cu Kα radiation (λ = 0.154056 nm), operated at 40 kV, 40 mA. The Raman spectra in the range 2000–100 cm^−1^ were obtained using an Almega dispersive Raman system (Nicolet) and a Nd:YAG intracavity doubled laser operating at 532 nm with an incident power of 10 mW. The X-ray photoelectron spectra (XPS) characterization was tested on an ESCA spectrometer (S-Probe ESCA SSX-100S, Fisons Instrument) and monochromatized Al Kα X-ray irradiation. The binding energy was calibrated by using the containment carbon (C 1s = 284.6 eV). The UV–vis diffuse reflectance spectra were examined using a UV–vis near-infrared spectrometer (UV-3150, Shimadzu). The low temperature nitrogen sorption measurement was conducted at 77 K using a Nova 3000e (Quantachrome Instruments, USA) with Quantachrome V11.0 software. The Brunauer–Emmett–Teller (BET) approach using adsorption data was adopted to determine the specific surface area. The sample was degassed at 150 °C for 20 h to remove physisorbed gases prior to the measurement. The ambient photoluminescence (PL) emission spectra characterizations were carried out on a fluorescence spectrophotometer (HITACHI F-4500) with an excitation wavelength of 360 nm.

### Photocatalytic activity

The photocatalytic activity of the TGN and branched TGN powders were evaluated by photodegradation of phenol in water under UV light illumination. In a typical procedure, 25 mg of the photocatalyst was added to 50 mL of 10 ppm phenol aqueous solution. The suspension was stirred in the dark for 1 h to reach an adsorption–desorption equilibrium, which was then subjected to the UV-irradiation with an intensity of ≈7.3 mW/cm^2^. 0.4 mL of the solution was collected and filtered to record the change of phenol concentration every hour, which was monitored using a liquid chromatography apparatus (Wufeng LC100, WondaCract ODS-2 column, China).

## Supporting Information

File 1 Additional FESEM images, low-temperature N_2_ adsorption isotherms and photodegradation results to support the discussion.
