# Supplementary material for: Sheet-on-belt branched TiO2(B)/rGO powders with enhanced photocatalytic activity
Source: Beilstein J Nanotechnol. 2018 May 24;9:1550–7. doi: 10.3762/bjnano.9.146 (PMC6009680; doi:10.3762/bjnano.9.146)
Supplement: File 1 — Additional FESEM images, low-temperature N2 adsorption isotherms and photodegradation results to support the discussion. [file Beilstein_J_Nanotechnol-09-1550-s001.pdf]

Supporting Information
for
Sheet-on-belt branched TiO_2(B)/rGO powders with enhanced photocatalytic activity

Huan Xing[1], Wei Wen[1,2] and Jin-Ming Wu[*1]

Address: [1]State Key Laboratory of Silicon Materials, and School of Materials Science and Engineering, Zhejiang University, Hangzhou 310027, P. R. China and [2]College of Mechanical and Electrical Engineering, Hainan University, Haikou 570228, P. R. China

Email: Jin-Ming Wu - msewjm@zju.edu.cn

* Corresponding author

Additional FESEM images, low-temperature N_2 adsorption isotherms and photodegradation results to support the discussion

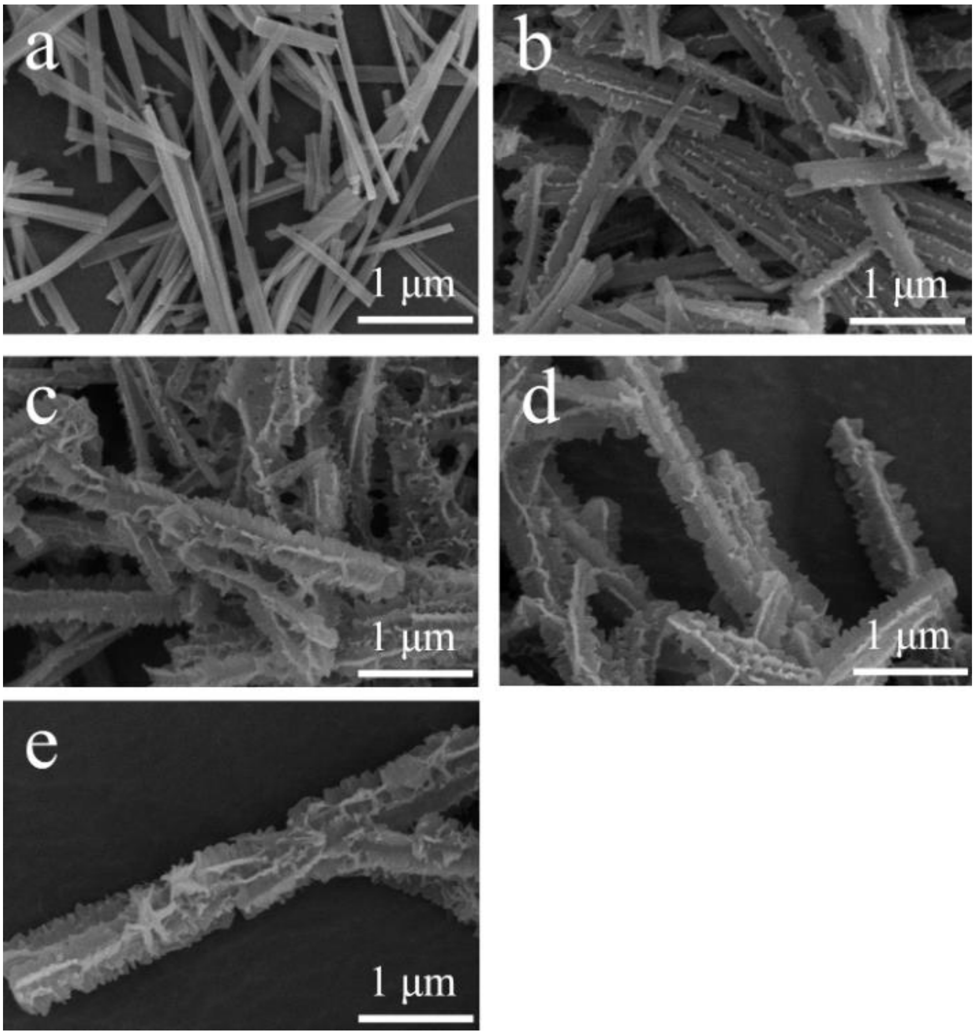

Figure S1: FESEM images of TGN-branches achieved by immersing in the precursor solution for (a) 0 h, (b) 2 h, (c) 4 h, (d) 6 h and (e) 8 h.

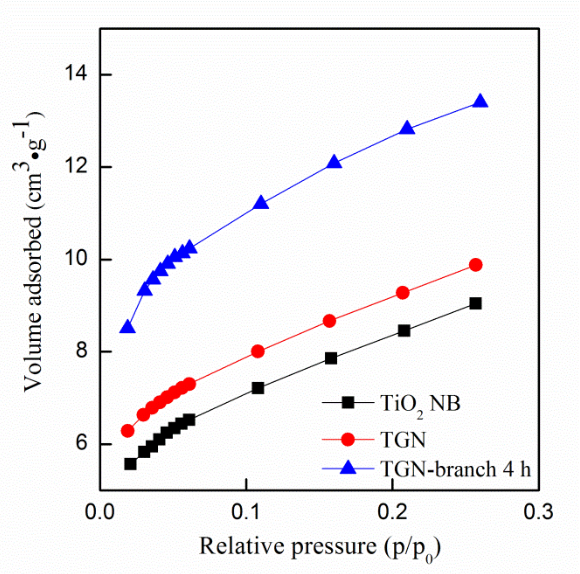

Figure S2: The low-temperature N_2 adsorption isotherms of the pristine TiO_2 nanobelt (TiO_2 NB), TGN and TGN-branch 4 h.

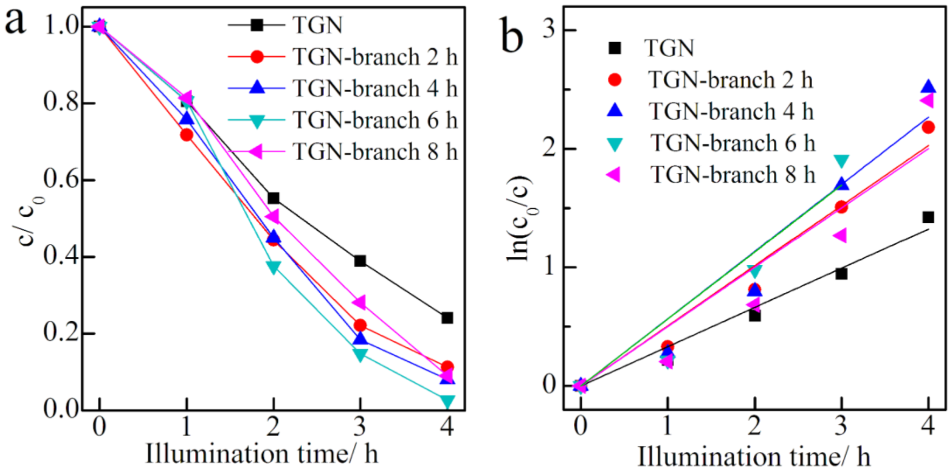

Figure S3: Photodegradations of phenol in the presence of TGN and TGN-branches achieved by immersing in the precursor solution for various durations, under UV light illumination: (a) the degradation curves; (b) the fitting results assuming a pseudo-first order reaction.
